# Subcellular Localization of Class I Histone Deacetylases in the Developing *Xenopus* tectum

**DOI:** 10.3389/fncel.2015.00510

**Published:** 2016-01-12

**Authors:** Xia Guo, Hangze Ruan, Xia Li, Liming Qin, Yi Tao, Xianjie Qi, Juanmei Gao, Lin Gan, Shumin Duan, Wanhua Shen

**Affiliations:** ^1^Zhejiang Key Laboratory of Organ Development and Regeneration, College of Life and Environmental Sciences, Hangzhou Normal UniversityHangzhou, China; ^2^Key Laboratory of Medical Neurobiology of Ministry of Health of China, Key Laboratory of Neurobiology, Department of Neurobiology, Zhejiang University School of MedicineHangzhou, China; ^3^Department of Neurosurgery, First Affiliated Hospital of Nanjing Medical UniversityNanjing, China

**Keywords:** histone deacetylases, mitochondria, *Xenopus laevis*, histone deacetylase inhibitors, subcellular localization, histone deacetylase 1

## Abstract

Histone deacetylases (HDACs) are thought to localize in the nucleus to regulate gene transcription and play pivotal roles in neurogenesis, apoptosis, and plasticity. However, the subcellular distribution of class I HDACs in the developing brain remains unclear. Here, we show that HDAC1 and HDAC2 are located in both the mitochondria and the nucleus in the *Xenopus laevis* stage 34 tectum and are mainly restricted to the nucleus following further brain development. HDAC3 is widely present in the mitochondria, nucleus, and cytoplasm during early tectal development and is mainly distributed in the nucleus in stage 45 tectum. In contrast, HDAC8 is broadly located in the mitochondria, nucleus, and cytoplasm during tectal development. These data demonstrate that HDAC1, HDAC2, and HDAC3 are transiently localized in the mitochondria and that the subcellular distribution of class I HDACs in the *Xenopus* tectum is heterogeneous. Furthermore, we observed that spherical mitochondria accumulate in the cytoplasm at earlier stages, whereas elongated mitochondria are evenly distributed in the tectum at later stages. The activity of histone acetylation (H4K12) remains low in mitochondria during tectal development. Pharmacological blockades of HDACs using a broad spectrum HDAC inhibitor of Trichostatin A (TSA) or specific class I HDAC inhibitors of MS-275 and MGCD0103 decrease the number of mitochondria in the tectum at stage 34. These findings highlight a link between the subcellular distribution of class I HDACs and mitochondrial dynamics in the developing optic tectum of *Xenopus laevis*.

## Introduction

The classical HDAC family consists of four classes (class I, IIa, IIb, and IV), which are highly conserved from invertebrates to mammals (Haberland et al., 2009). The 11 HDACs play various roles in neural development (Akhtar et al., 2009; Montgomery et al., 2009), synaptic plasticity (Gräff and Tsai, 2013), and neurological disease (Haberland et al., 2009). Class I HDACs have traditionally been reported to be localized to the nucleus via a nuclear localization signal (NLS) and to function as transcriptional repressors (de Ruijter et al., 2003; Haberland et al., 2009). However, there is considerable debate over the subcellular distribution of class I HDACs in the nervous system. HDAC1 and HDAC2 are restricted to the nucleus because they lack the nuclear export sequence (NES) (Gräff and Tsai, 2013). HDAC1 and HDAC3 can also be sequestered in the cytoplasm by association with IκBα in cultured cells (Viatour et al., 2003). Some studies demonstrated that HDAC1 or HDAC2 can be translocated from the nucleus to the cytoplasm by dissociation of the CoREST/REST/HDAC1 repressor complex in virus-infected cells (Gu et al., 2005). In general, previous studies paid more attention to the distribution and movement of HDACs between the nucleus and the cytoplasm. Therefore, the subcellular localization and function of class I HDACs have yet to be studied in detail.

Mitochondria not only provide metabolic energy for cell survival but also regulate neurogenesis (Chung et al., 2007; Voloboueva et al., 2010; Oruganty-Das et al., 2012; Steib et al., 2014), neuroplasticity (Cheng et al., 2010), apoptosis (Desagher and Martinou, 2000; Karbowski et al., 2002), and neurodegeneration (Chen et al., 2007; Chen and Chan, 2009; Guedes-Dias and Oliveira, 2013). The distribution and trafficking of mitochondria play crucial roles in maintaining proper neuronal structure and function (Li et al., 2004; Saxton and Hollenbeck, 2012). The number and shape of mitochondria are maintained by the balance of mitochondrial fission and fusion events (Cerveny et al., 2007; Youle and van der Bliek, 2012). Disruption of such balance results in mitochondrial elongation or fragmentation (Cerveny et al., 2007). Dynamics of mitochondrial fission and fusion are regulated by mitochondria-related proteins, including the fission-related proteins FisI (Yoon et al., 2003), dynamin-related protein 1 (Drp I) (Smirnova et al., 2001) and the fusion-related proteins mitofusion 1 (Mfn1), mitofusion 2 (Mfn2) (Chen et al., 2003), and optic atrophy 1 (Opa I) (Olichon et al., 2003). Previous studies have focused on the mechanism of the transition from an elongated to a punctiform phenotype of mitochondria during cell death or apoptosis (Desagher and Martinou, 2000; Frank et al., 2001; Detmer and Chan, 2007). However, there is a lack of evidence regarding mitochondrial morphology and dynamics during brain development.

In the present study, we present multiple lines of evidence that in the developing *Xenopus* tectum, class I HDACs are transiently expressed in the mitochondria at earlier stages and are exported to the nucleus or cytoplasm at later stages. The subcellular distribution of class I HDACs is heterogeneous. Furthermore, we observed that mitochondria are dynamic in the developing tectum. The number of mitochondria is mainly regulated by class I HDACs. These data describe the developmental regulation of the localization of class I HDACs in the mitochondria and their role in establishing mitochondrial morphology in the developing intact vertebrate brain.

## Materials and methods

### Animals and rearing

All animal procedures were performed according to the requirements of the “Regulation for the Use of Experimental Animals in Zhejiang Province.” This study has been approved by the local ethics committee of the Hangzhou Normal University. Tadpoles were obtained by the mating of adult *albino Xenopus* injected with human chorionic gonadotropin (HCG) and raised on a 12 h dark/light cycle in Steinberg's solution [(in mM): 10 HEPES, 58 NaCl, 0.67 KCl, 0.34 Ca(NO_3_)_2_, 0.83 MgSO_4_, pH 7.4] in a 20°C incubator. Tadpoles were anesthetized in 0.02 % MS-222 (3-aminobenzoic acid ethyl ester methanesulfonate, Sigma-Aldrich) for experimental manipulations. Under our rearing conditions, tadpoles reached stage 44–46 at 6–7 days post-fertilization (dpf) and stage 48–49 at 8–11 dpf. Tadpole stages were determined according to significant developmental changes in the anatomy (Nieuwkoop and Faber, 1956).

### Drugs and treatment

To block the histone deacetylase activity, tadpoles were incubated with TSA (Sigma-Aldrich) (Tseng et al., 2011), MS-275 or MGCD0103 (Selleck) (Bolden et al., 2006; Bradner et al., 2010) in Steinberg's solution for 24–48 h.

### Immunohistochemistry and image analysis

Tadpoles were anesthetized in 0.02% MS-222, and fixed in 4% paraformaldehyde (PFA, pH 7.4) at 4°C overnight. Tadpoles were rinsed with 0.1 M phosphate buffer (PB, pH 7.4) and immerged in 30% sucrose overnight for dehydration. On the second day, animals were embedded in optimal cutting temperature (OCT) media, and cut into 20 μm cryostat sections with a microtome (Microm, HM550 VP). Sections were rinsed with 0.1 M PB for 2 X 20 min, and permeabilized with 0.3% Triton X-100 in PB, and blocked in 5% donkey serum for 1 h before incubating with primary antibodies at 4°C overnight. For primary antibodies, we used the antibodies of anti-HDAC1 (1:200, Rabbit, Abcam, ab33278), anti-HDAC2 (1:200, Rabbit, Abcam, ab137364), anti-HDAC3 (1:200, Rabbit, Abcam, ab16047), anti-HDAC8 (1:200, Rabbit, Abcam, ab137474), anti-COX IV (cytochrome c oxidase subunit IV, 1:200, Rabbit, Abcam, ab33985), and anti-H4K12 (Histone H4 acetyl K12, 1:500, Rabbit, Abcam, ab46983). Sections were rinsed with PB and incubated with secondary antibody (FITC or Rhod or Alexa 647) for 1 h at room temperature. After sections were counterstained with DAPI, mounted and sealed, the immunofluorescent images were collected using a Zeiss LSM 710 confocal microscope. For mitochondria counterstaining, brain slides were immersed in 0.2 mM MitoTracker Green (Invitrogen, M7510) for 30 min and mounted on slides for imaging.

For each brain, six representative sections were collected for analysis. The first section was taken where the two tectal lobes meet at the midline of ventricular layer and the last section was taken where the anterior ventricle appears at the midline. The brain sections were scanned by confocal microscopy (LSM710, Zeiss, Germany) and analyzed by iMaris (Bitplane AG, Zurich) image processing software. Numbers of positive cells were counted using the mode of Surpass feature in iMaris. For each section, the region selected for cell number counting was delineated by the whole optic tectum. The same parameters for counting cells were used for all of the sections analyzed. All positive cells from the representative sections were added and compared by statistical analysis.

### Western blot and antibody specificity test

The dissected optical tecta or murine cerebral cortex were homogenized in the radioimmunoprecipitation assay (RIPA) buffer with a protease inhibitor cocktail (1:100, Sigma Aldrich) at 4°C. Protein homogenates were separated by SDS-PAGE and transferred to PVDF membranes. Membranes were blocked in 4% nonfat milk for 1 h with TBS buffer containing 0.1% Tween-20 (Sigma Aldrich) (TBST) and incubated with primary antibodies overnight at 4°C. The specificity of antibodies was performed using antibodies of anti-HDAC1 (1:1000), anti-HDAC2 (1:1000), anti-HDAC3 (1:1000), and anti-HDAC8 (1:500), which were diluted in 4% nonfat milk. Blots were rinsed with TBST and incubated with horseradish peroxidase (HRP)-conjugated secondary antibodies (1:2000, Invitrogen) for 1 h at room temperature. Bands were visualized using ECL chemiluminescence (1:1, Pierce).

### Electron microscopy

Tadpoles at stages 34 and 45 were deeply anesthetized with 0.02% MS222 in Steinberg's rearing solution and fixed in a mixture of 2% paraformaldehyde, 2% glutaraldehyde, and 0.02% CaCl_2_ in 0.035 M sodium cacodylate buffer at pH 7.4. Tadpole brains were dissected and immersed in fixative for 1 week at 4°C. Tecta were rinsed in 0.1 M phosphate-buffered saline (PBS, pH 7.4) and post-fixed in 2% osmium tetroxide for 1 h. Brains were dehydrated in an acetone series (50, 70% with 1% uranyl acetate, 90, 100%) and infiltrated with Epon 812 resin (50, 75, and 100% in acetone respectively; Tedpella Inc, Tustin Calif.). On the next day, the brains were flat embedded in 100% Epon 812 resin between two sheets of Aclar plastic (Ladd Research, Williston, VT) and incubated at 65°C for 48 h. Seventy nanometer-thin sections were prepared using ultrathin microtome (UC6, Leica, Germany) and mounted on 200-mesh formvar-coated copper grids (Plano, Wetzlar, Germany). Images were acquired with H-7650 (Japanese Electronic Company) electron microscopy. Mitochondrial size were outlined with magnetic lasso and analyzed by image analysis tool in Adobe Photoshop.

### Statistics

Paired data were tested with Student's *t*-test. Multiple group data were tested with an ANOVA followed by *post-hoc* Tukey's test unless noted. Data are represented as mean ± SEM. Experiments and analysis were performed blind to the experimental condition unless noted.

## Results

### HDAC1 localization is developmentally regulated during early development of the *Xenopus tectum*

**Figure 1:**
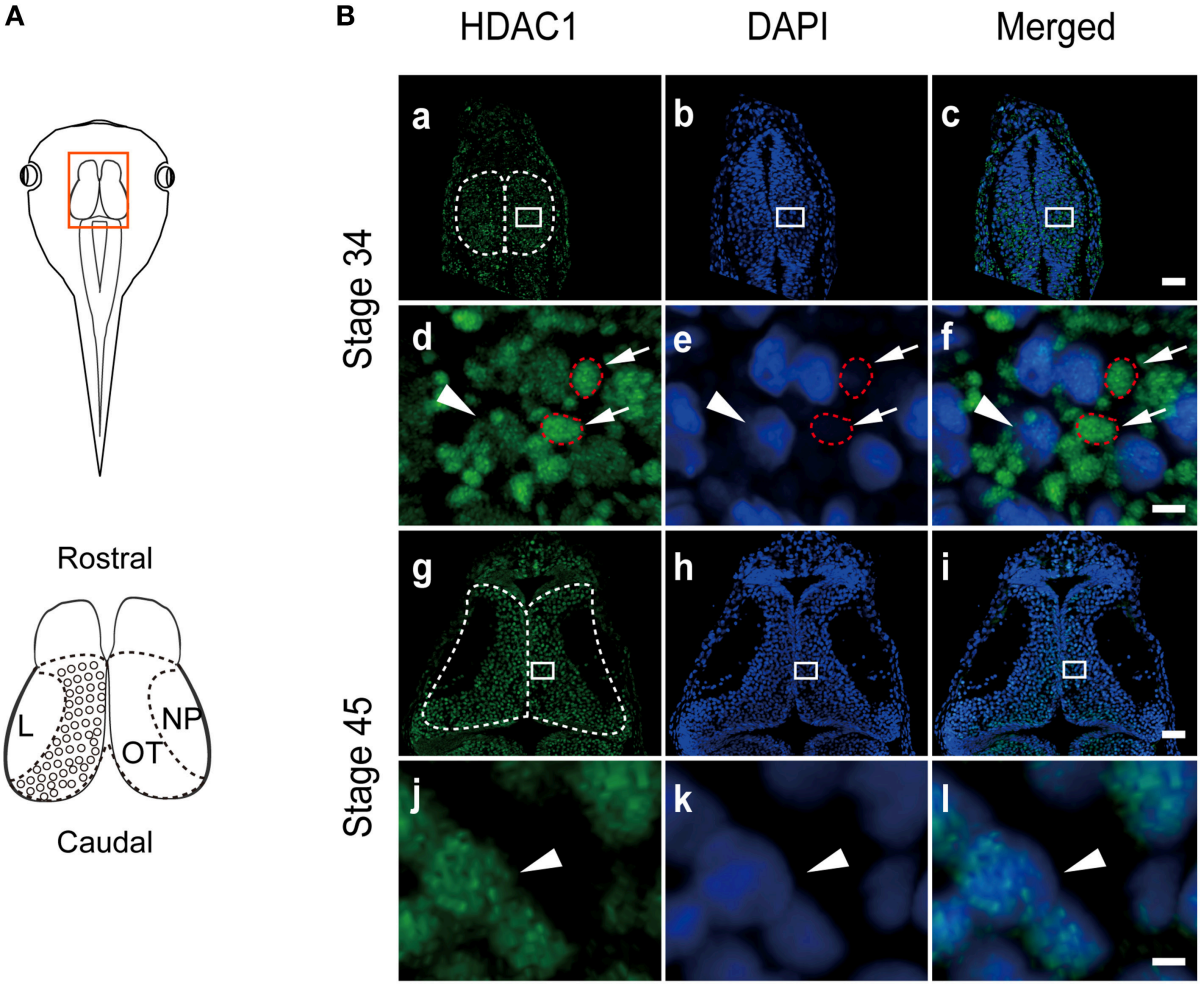
**HDAC1 distribution in tectal cells in the developing *Xenopus*. (A)** A cartoon showing the optic tectum at stage 45. The tectum is outlined in red. The brain was fixed and cryosectioned for immunostaining. NP, neuropil; OT, optic tectum; L, lateral. **(B)** The tectum was sectioned and stained with anti-HDAC1 antibody (green) at stage 34 **(Ba–Bc)**. Scale bar: 50 μm. A higher-magnification image of the tectum is shown below. Scale bar, high magnification: 5 μm. HDAC1 was clearly localized in the organelle showing weak DAPI staining (**Bd–Bf**, red circles), whereas little HDAC1 was distributed in the DAPI-positive cell nuclei (arrowhead). The subcellular localization of HDAC1 in the organelle was almost absent from the tectum at stage 45 **(Bg-Bl)**. Arrowheads indicate cell nuclei counterstained with DAPI **(Bg-Bl)**.

HDAC family members are highly conserved between mammals and vertebrates (Gräff and Tsai, 2013). The amino acid alignment of HDAC1 between *Homo sapiens* and *Xenopus laevis* showed that the orthologs of class I HDACs share 71–97% protein sequence homology (Figure S1). Previous studies showed that HDAC1 is a transcriptional repressor that is mainly located in the nucleus (Kim et al., 2010). However, the subcellular distribution of HDACs in early brain development remains unclear. To determine the distribution of HDAC1 in vertebrates, we immunostained the optic tectum in developing *Xenopus laevis* (Figure 1A) using a specific anti-HDAC1 antibody (Figure S2A), which was raised against human HDAC1 (amino acids 50-161) (Figure S1A, underline). Surprisingly, we observed that HDAC1 was not restricted to the nucleus at stage 34 (Figures 1Ba–c). Interestingly, HDAC1-positive clusters were localized primarily to the cytoplasm (Figures 1Ba–f) and restricted to the nucleus at stage 45 (Figures 1Bg–l). Furthermore, enhancement of counterstained DAPI signaling revealed co-localization between HDAC1 clusters and DAPI staining (Figure 1Bf). Because mitochondrial DNA (mtDNA) can be stained by DAPI (Spelbrink et al., 2001), this result suggests that HDAC1 may be localized in the mitochondria. These data indicate that subcellular localization of HDAC1 is developmentally regulated in the developing tectum.

### Subcellular localization of HDAC1

**Figure 2:**
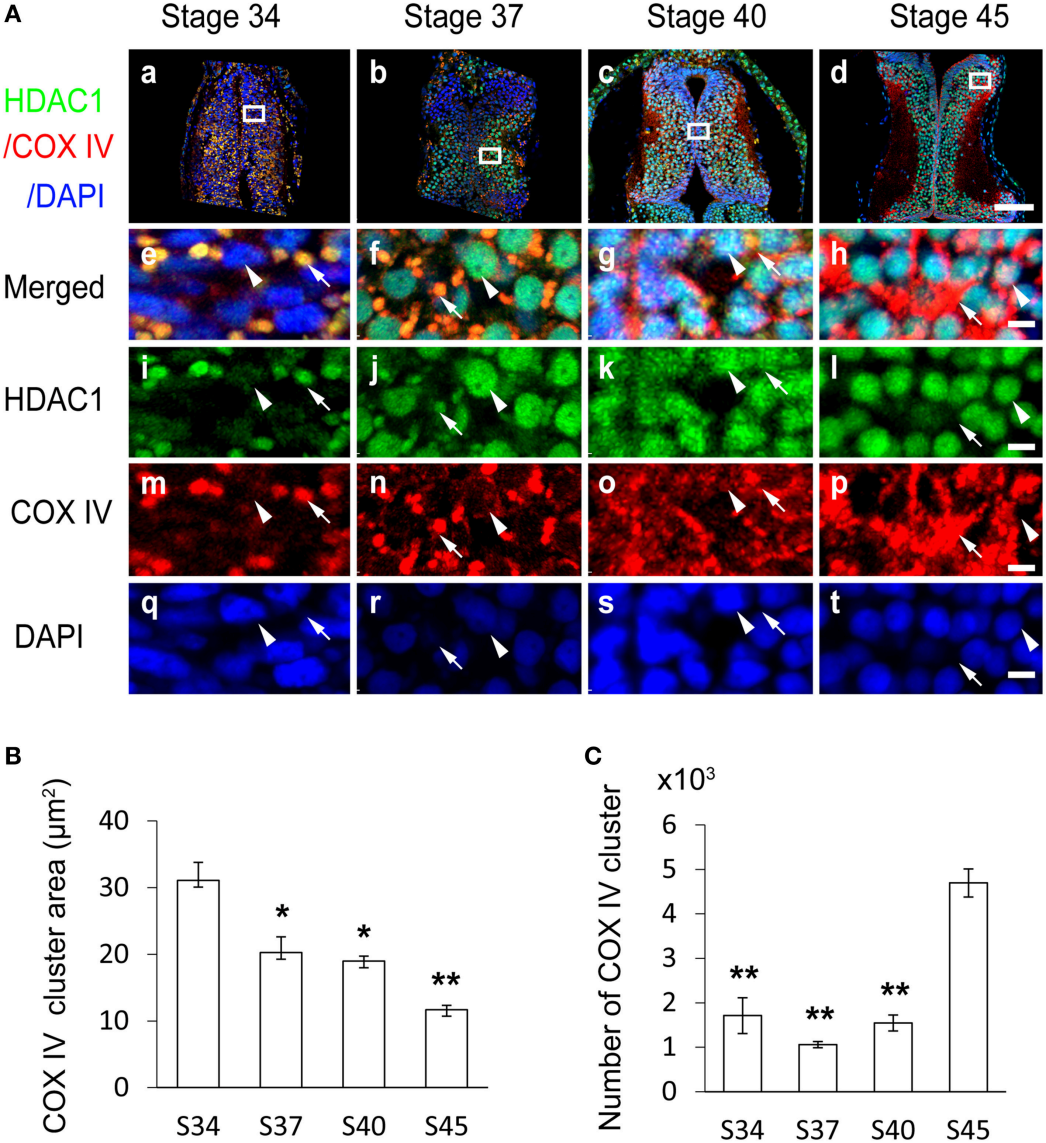
**Subcellular distribution of HDAC1 in the developing *Xenopus* tectum. (A)** Representative immunofluorescent images showing merged HDAC1 (green)/COX IV (red)/DAPI (blue) staining in cells of the developing tectum at stages 34 **(Aa)**, 37 **(Ab)**, 40 **(Ac)**, and 45 **(Ad)**. Scale bar: 100 μm. High-magnification images (**Ae–At**, scale bar: 5 μm) are demarked by white lines and connected to the original figures **(Aa–Ad)**. Arrowheads indicate cell nuclei stained with DAPI, and arrows indicate mitochondria stained for COX IV. HDAC1 distribution is represented by anti-HDAC1 staining. **(B)** Summary showing that area of mitochondria stained by anti-COX IV antibody was decreased in tadpoles at stages 37, 40, and 45, during the development of the tectum, compared to stage 34 tadpoles. **(C)** Quantification showing that the number of mitochondria in the tectum was significantly increased at stage 45 compared to stages 34, 37, and 40. *N* = 3, 3, 3, 3 for stage 34, 37, 40, and 45. ^*^p < 0.05, ^**^p < 0.01.

To further assess the organelles containing HDAC1 in tadpoles at stage 34, we co-immunostained the tectum with the anti-HDAC1 antibody (Figure S1A) and an anti-COX IV antibody that is a specific marker for mitochondria (Figure S3). We found that the HDAC1 and COX IV staining were co-localized in stage 34 tadpoles (Figures 2Aa,e,i,m,q). To test whether the subcellular distribution of HDAC1 changed with the maturation of the tectum, we used an anti-HDAC1 antibody to immunostain cryosections of the optic tectum at stages 34, 37, 40, and 45 (Figures 2Aa–t). Brain sections were scanned with a confocal microscope. We observed that HDAC1 was mainly localized to the mitochondria rather than to the nucleus at stage 34 (Figure 2Ae and Table 1), and it was mostly excluded from mitochondria at stage 45 (Figure 2Al). The HDAC1 in mitochondria gradually decreased during the maturation of tectum (Figure 2A and Table 1), whereas HDAC1 in the nucleus was dramatically up-regulated (Figures 2Ai–l). By the time tadpoles reached stages 40–45, HDAC1 was mainly confined to the cell nuclei (Figure 2A and Table 1).

**Table 1:** **Subcellular distribution of HDAC1, HDAC2, HDAC3, and HDAC8 in the cytoplasm, nucleus, and mitochondria during tectal development**.

	**Stage 34**			**Stage 37**			**Stage 40**			**Stage 45**		
**Protein**	**C**	**N**	**M**	**C**	**N**	**M**	**C**	**N**	**M**	**C**	**N**	**M**
HDAC1	−	+	+++	−	+++	+	−	+++	+	−	+++	−
HDAC2	−	++	+	−	++	+	−	+++	+	−	+++	−
HDAC3	+	++	+	+	++	+	+	++	+	−	+++	−
HDAC8	++	+	+	++	+	+	++	+	+	++	+	+

*Subcellular location of HDAC1, HDAC2, HDAC3, and HDAC8 in cytoplasm (C), nucleus (N), and mitochondria (M) was determined by immunohistochemistry. −, < 5%; +, ~30%; ++, ~50%; +++, ~80%*.

To further distinguish the specific cytoplasmic organelles with which HDAC1 was co-localized, we used anti-lamp1, anti-EEA1, and anti-calnexin antibodies to specifically label lysosomes, early endosomes, and endoplasmic reticulum in stage 47 tadpoles (Figure S3). We found that the spatial distribution of COX IV clusters differed from lamp1 (Figure S3A), EEA1 (Figure S3B), and calnexin (Figure S3C) clusters. Furthermore, the colocalization between HDAC1 and COX IV clusters was close to 100% (Figures 2Ae,f), indicating that the organelles containing HDAC1 are mitochondria.

To measure the developmental changes in mitochondrial dynamics, COX IV-positive clusters were counted with iMaris software (Figures 2B,C; see Materials and Methods for details). We observed that the mean area of COX IV-positive mitochondria decreased with the development of the tectum from stage 34 to stage 45 (Figure 2B). The number of mitochondria was maintained from stage 34 to stage 37 and drastically increased at stage 45 compared to earlier stages (Figure 2C).

**Figure 3:**
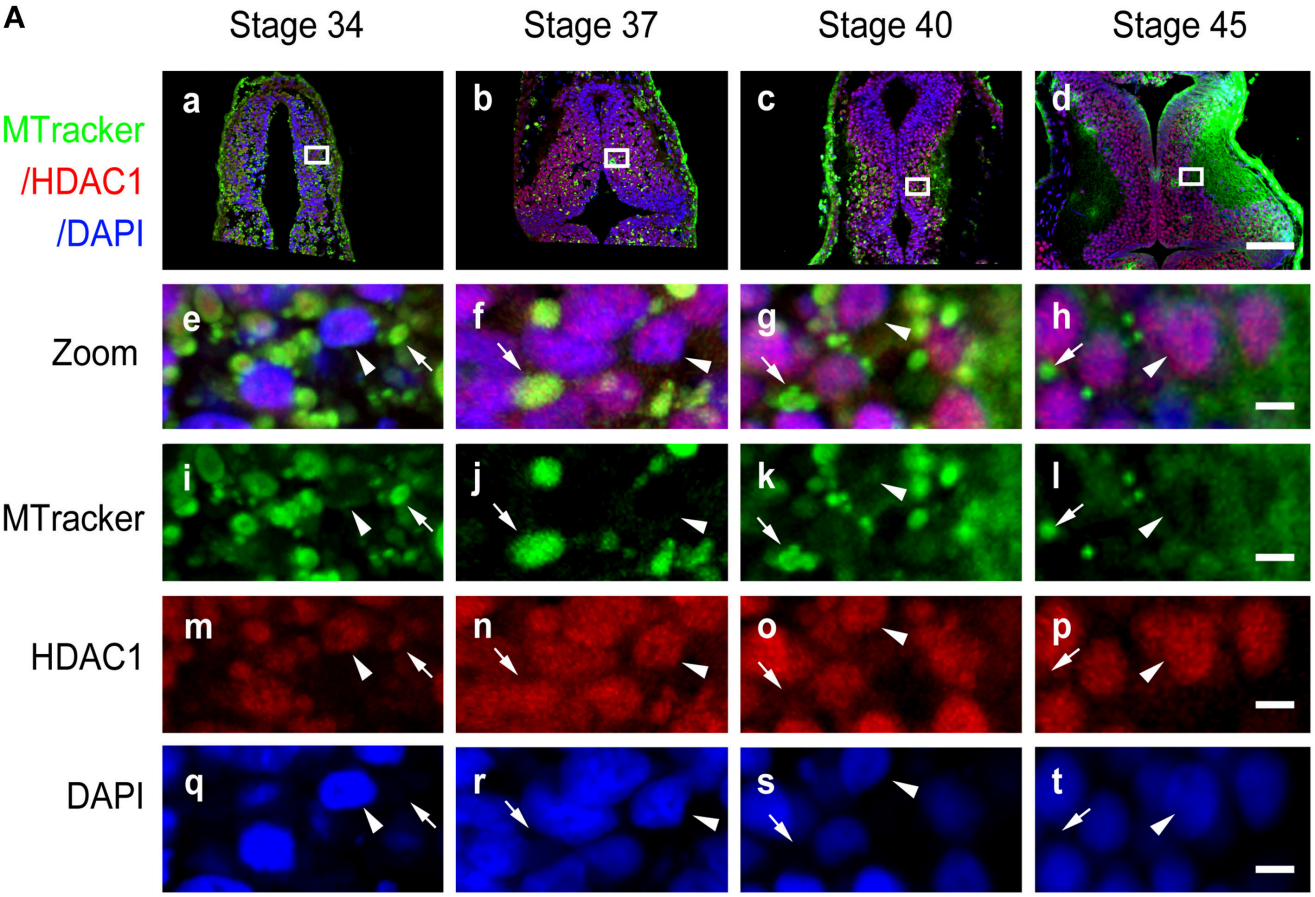
**Co-localization of HDAC1 and MitoTracker in developing tectum. (A)** Representative co-staining images showing the anti-HDAC1 antibody (red) and MitoTracker (MTracker, green) in stage 34 **(Aa)**, 37 **(Ab)**, 40 **(Ac)**, and 45 **(Ad)** tecta. Scale bar: 100 μm. High-magnification images (**Ae–At**, scale bar: 5 μm) are demarked by white lines from the original figures **(Aa-Ad)**. Arrowheads indicate cell nuclei stained with DAPI, whereas arrows indicate mitochondria stained with MitoTracker. Scale bar: 5 μm.

To further test the subcellular localization of HDAC1, we used a specific mitochondrial marker, MitoTracker green (Figure 3A), to label mitochondria after HDAC1 staining in the optic tectum (Figures 3Aa–t). In line with our previous observations (Figure 2A), HDAC1 was mainly retained in the mitochondria in stage 34/37 tadpoles and was located in the nuclei in stage 40/45 tadpoles (Figure 3Aa). These results indicate that HDAC1 is mainly distributed in the mitochondria during early tectal development and localized in the nuclei following the maturation of the tectum.

### Ultrastructural changes in mitochondrial morphology

**Figure 4:**
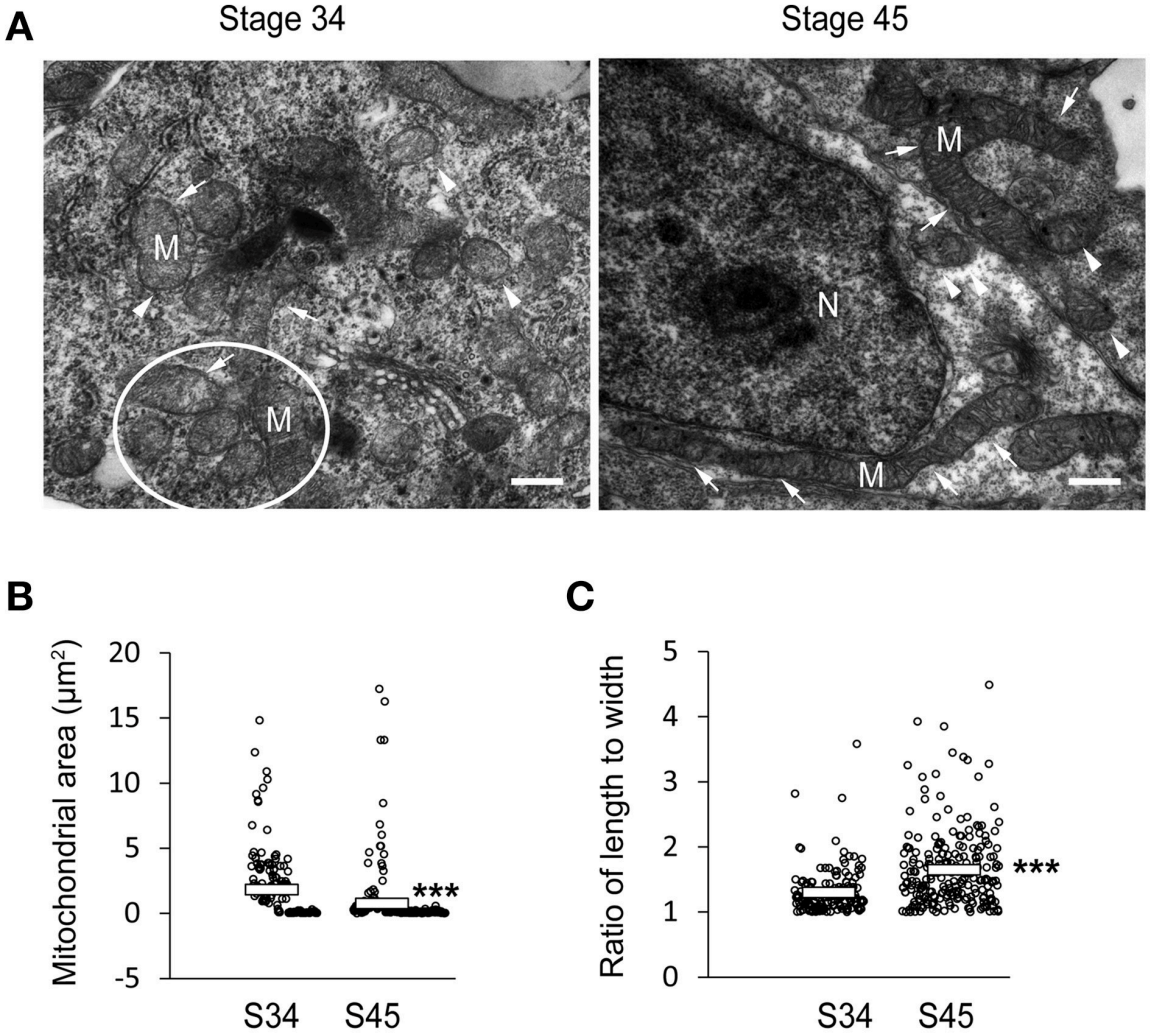
**Mitochondrial morphology changes during the development of tectal cells. (A)** Tecta at stages 34 (left) and 45 (right) were fixed for electron microscopy. White circle indicate aggregated mitochondria. Arrows indicate elongated mitochondria, whereas arrowheads indicate round/spherical mitochondria. Scale bar: 0.5 μm. M, mitochondria; N, Nucleus. **(B)** Quantification showing that the area of mitochondria in tecta was significantly decreased at stage 45 compared to stage 34 (Stage 34: 1.82 ± 0.20 μm^2^, Stage 45: 0.75 ± 0.15 μm^2^; *N* = 161, 224). **(C)** The ratio of mitochondrial length to width was dramatically increased at stage 45 compared to stage 34 (Stage 34: 1.30 ± 0.03, Stage 45: 1.65 ± 0.04; *N* = 161, 224). ^***^p < 0.001.

**Figure 5:**
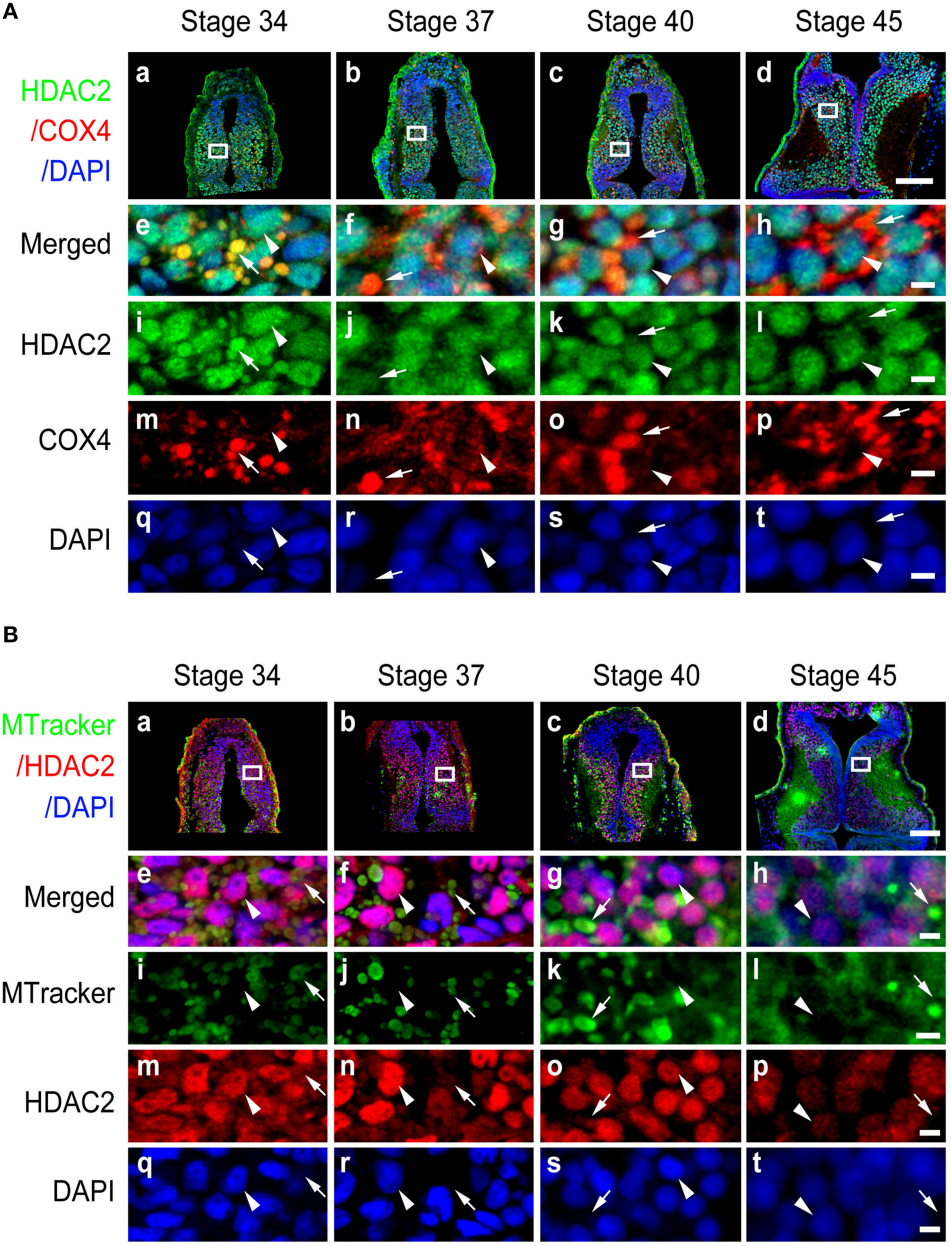
**Subcellular distribution of HDAC2 in developing tectum. (A)** Immunostaining for HDAC2 (green)/COX IV (red)/DAPI (blue) in the developing tectum at stages 34 **(Aa)**, 37 **(Ab)**, 40 **(Ac)**, and 45 **(Ad)**. Scale bar: 100 μm. High-magnification images (**Ae–At**, Scale: 5 μm) are demarked by white lines and are connected to the original figures **(Aa-Ad)**. Arrowheads indicate cell nuclei stained with DAPI, whereas arrows indicate mitochondria stained for COX IV. **(B)** Tectal cells were stained for HDAC2 (red) with an anti-HDAC2 antibody and counterstained with MitoTracker green at stages 34 **(Ba)**, 37 **(Bb)**, 40 **(Bc)**, and 45 **(Bd)**. Scale bar: 100 μm. High-magnification images (**Be–Bt**, scale bar: 5 μm) are demarked by white lines and are connected to the original figures **(Ba–Bd)**. Arrowheads indicate cell nuclei stained with DAPI, whereas arrows indicate mitochondria stained with MitoTracker.

**Figure 6:**
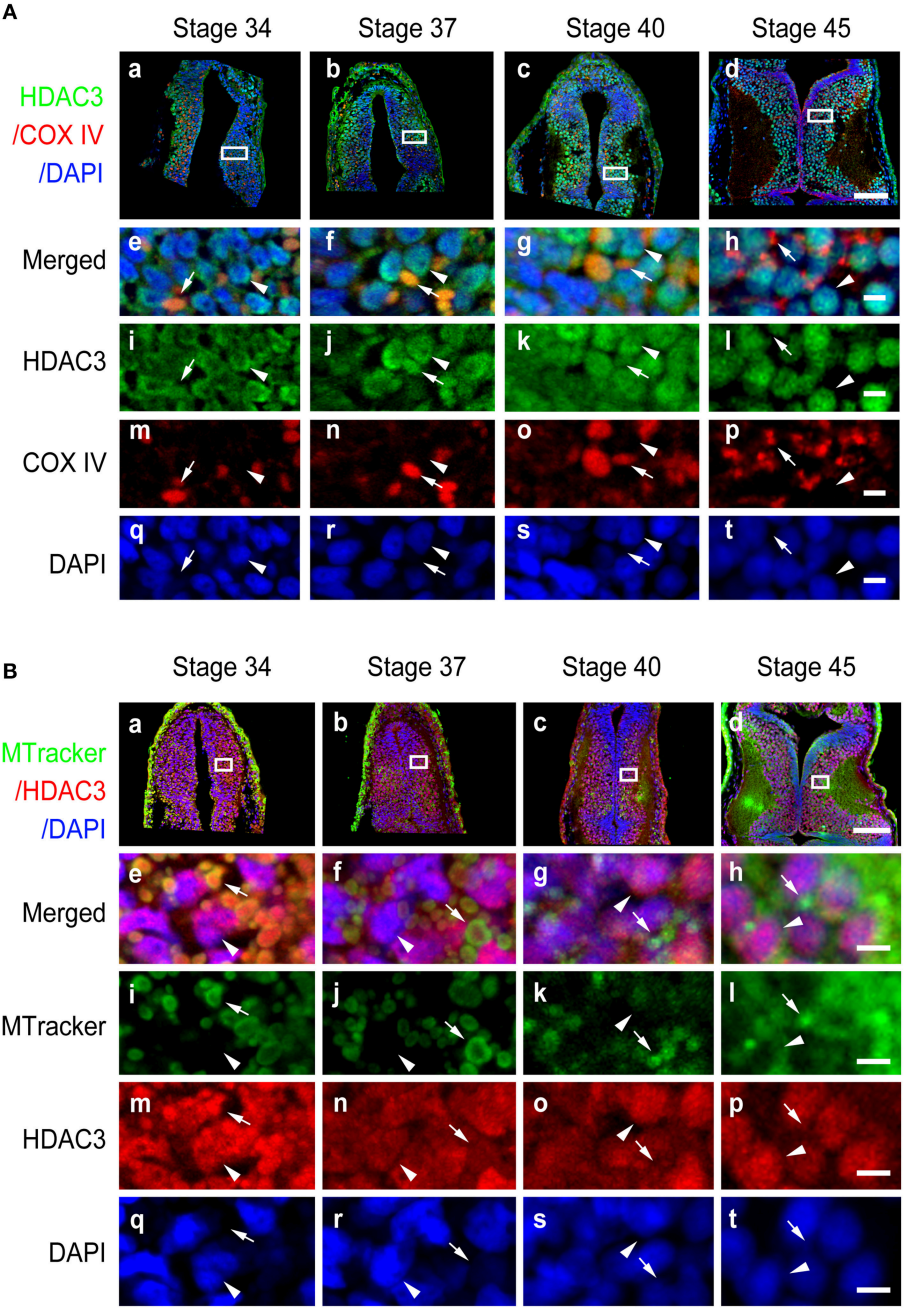
**Subcellular distribution of HDAC3 in the developing tectum. (A)** Representative immunofluorescent images showing merged HDAC3 (green)/COX IV (red)/DAPI (blue) staining in cells of the developing tectum at stages 34 **(Aa)**, 37 **(Ab)**, 40 **(Ac)**, and 45 **(Ad)**. Scale bar: 100 μm. High-magnification images (**Ae–At**, scale bar: 10 μm) are demarked by white lines and are connected to the original figures **(Aa-Ad)**. **(B)** Tecta at stages 34 **(Ba)**, 37 **(Bb)**, 40 **(Bc)**, and 45 **(Bd)** were stained for HDAC3 (red) with an anti-HDAC3 antibody and counterstained with MitoTracker green. Scale bar: 100 μm. High-magnification images (**Be–Bt**, scale bar: 5 μm) are demarked by white lines. Arrowheads indicate cell nuclei counterstained with DAPI, whereas arrows indicate mitochondria stained with MitoTracker.

**Figure 7:**
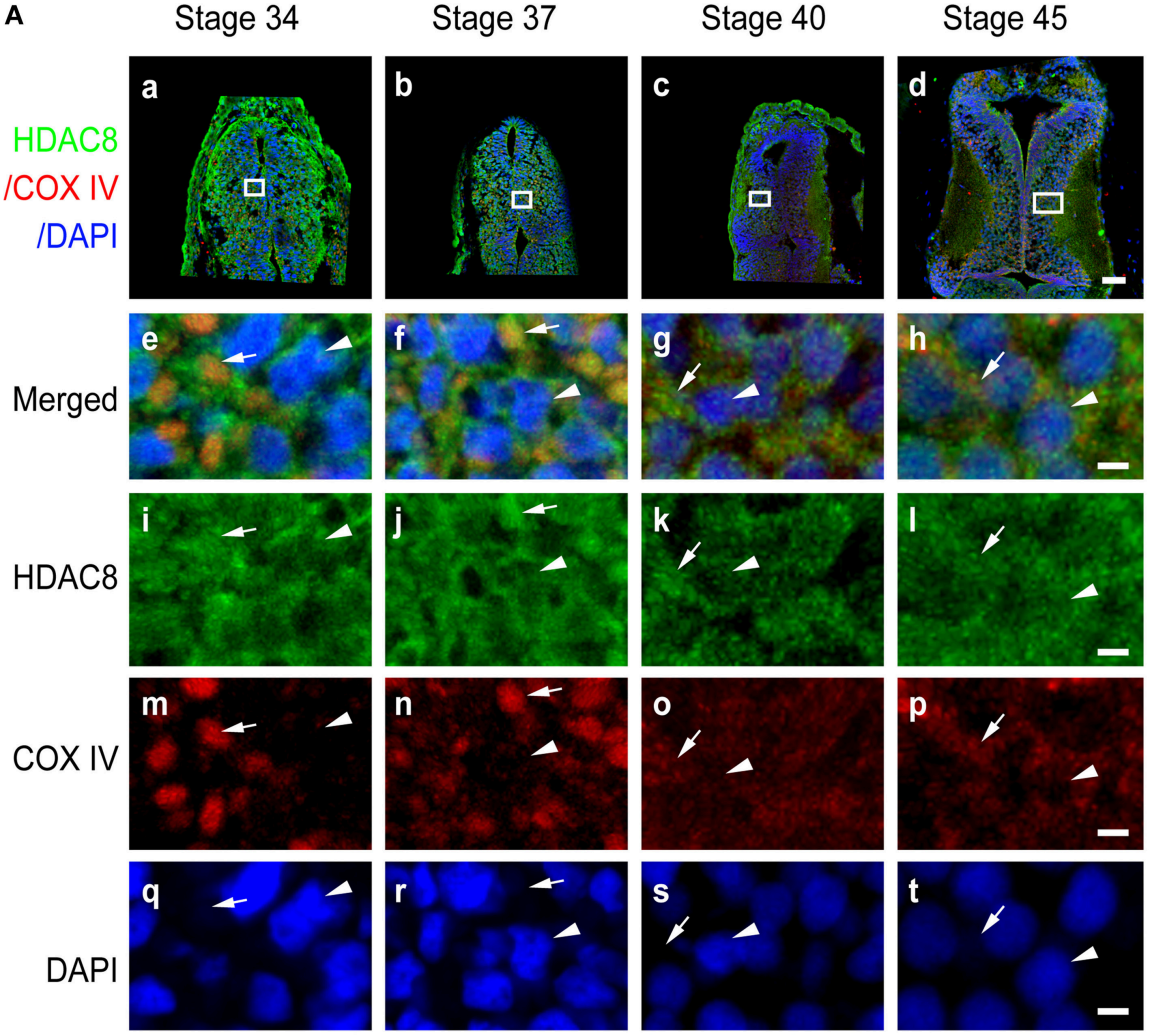
**Subcellular distribution of HDAC8 in the developing tectum. (A)** Sections of tectum were immunostained for HDAC8 (green), COX IV (red), and DAPI (blue) at stages 34 **(Aa)**, 37 **(Ab)**, 40 **(Ac)**, and 45 **(Ad)**. Scale bar: 50 μm. High-magnification images (**Ae–At**, scale bar: 5 μm) are demarked by white lines. Arrowheads indicate DAPI-stained cell nuclei, and arrows indicate COX IV-positive mitochondria.

We observed that the size of COX IV-positive clusters (Figure 2A) and MitoTracker positive staining (Figure 3A) decreased with tectal development. To further test whether these clusters were composed of single or aggregated mitochondria, we fixed tadpoles at stages 34 and 45 and performed electron microscopy. The optic tectum was sectioned, and mitochondria were imaged using electron microscopy. The EM analysis revealed that most of the mitochondria were rounded and short in tectal cells at stage 34, whereas they were thin and elongated (>10 μm) in tectal cells at stage 45 (Figure 4A). The mitochondria at stage 34 tended to be aggregated, consistent with the finding that COX IV-positive clusters in the tectum were larger at earlier developmental stages (Figures 2, 5A, 6A, 7A). The ultrastructure data were also consistent with the results of MitoTracker labeling, which showed that the size of mitochondria (Figure 4B) decreased with the development of tectum (Figures 3, 5B, 6B). The ratio of mitochondrial length to width was significantly increased in stage 45 tecta compared to stage 34 tecta (Figure 4C), suggesting that the mitochondria tended to elongate with the development of brain. These data indicate that the mitochondrial morphology is dynamic during early brain development.

### Subcellular distribution of HDAC2, HDAC3, and HDAC8

To test whether other class I HDAC family members displayed similar tempo-spatial distributions to that of HDAC1, we used specific anti-HDAC2 (Figure S2B), anti-HDAC3 (Figure S2C), and anti-HDAC8 (Figure S2D) antibodies to immunostain the optic tectum as described above. The peptide sequences for raising these antibodies are shown in Figures S1B,C. The specificity of the antibodies was performed by Western blot using *Xenopus* tecta and murine brain (Figure S2). We observed that HDAC2 was localized in both the nucleus and mitochondria at stage 34 (Figure 5Aa) and that HDAC2 was almost excluded from mitochondria at stage 45 (Figure 5Ad). The co-staining results for anti-HDAC2 and MitoTracker confirmed that HDAC2 levels decreased in the mitochondria during tectal development, whereas HDAC2 levels in the nuclei remained at a high level from stage 34 to stage 45 (Figure 5B).

In contrast to HDAC1 and HDAC2, HDAC3 was widely distributed in the cytoplasm, mitochondria, and nuclei from stage 34 to 40 in the tectum (Figures 6Aa–c). When tadpoles reached stage 45, HDAC3 was restricted to the nuclei (Figure 6Ad). Counterstaining with MitoTracker after HDAC3 immunostaining further confirmed the developmental changes in the subcellular distribution of HDAC3. Notably HDAC8 in *Xenopus* lacks a region corresponding to the N-terminal segments of HDAC8 in human (Figure S1D). The molecular weight of HDAC8 in tectum is approximately 30 kD (Figure S2D). HDAC8, on the other hand, was largely restricted to the cytoplasm, although it was weakly localized in the mitochondria at the stages we tested (Figure 7A). Overall, the localization of class I HDACs is divergent and is developmentally regulated in the developing optic tectum.

### Developmental changes of acetylation and regulation of mitochondrial dynamics by HDAC inhibitors

**Figure 8:**
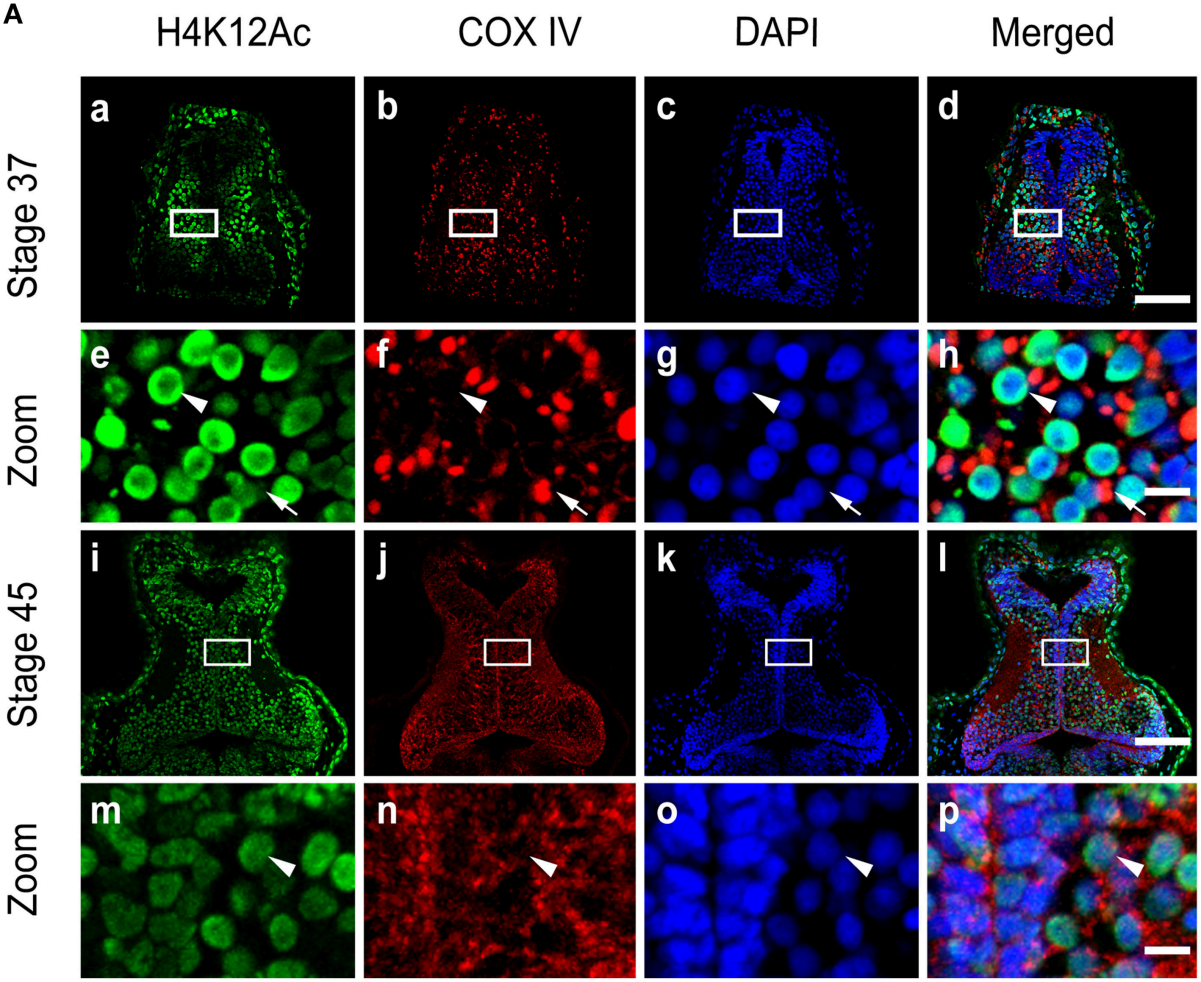
**Activity of acetylation in the mitochondria. (A)** Tecta at stages 37 **(Aa–Ah)** and 45 **(Ai–Ap)** were stained with antibodies against Histone H4 (acetyl K12, H4K12Ac) (green) and the mitochondrial marker COX IV (red). Scale bar: 100 μm. High-magnification images (**Ae–Ah**, **Am–Ap**, Scale bar: 10 μm) are demarked by white lines. Arrowheads indicate cell nuclei, and arrows indicate mitochondria.

The level of histone modification by H4K12 acetylation (H4K12Ac) is linked to active gene expression (Peleg et al., 2010). To test the histone acetylation, we performed immunohistochemistry on cryosections of stage 34 and 45 tecta using antibodies against acetylated H4K12. We observed that at stage 37, tectal cell nuclei maintained a basal level of histone acetylation of H4K12 in a subset of cells (Figures 8Aa–Ad) that was relatively high compared to the nuclei at stage 45 (Figures 8 Ai–l). Conversely, the levels of H4K12Ac in mitochondria remained low in the tectum at stage 37 (Figure 8Ah) and largely unchanged at stage 45 (Figure 8Ap).

**Figure 9:**
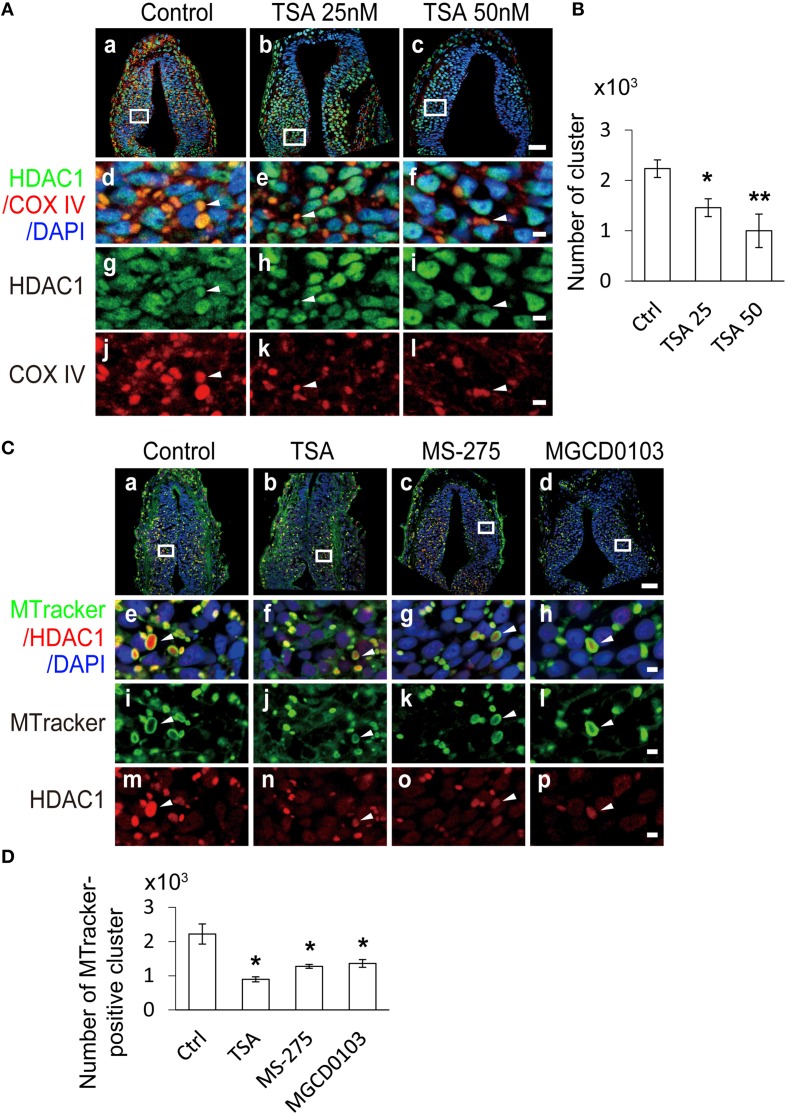
**Mitochondrial dynamics are regulated by HDAC inhibitors. (A)** Representative images showing changes in mitochondrial dynamics at stage 37 tadpoles. Tecta in the control **(Aa)**, TSA 25 nM **(Ab)**, and TSA 50 nM **(Ac)** treatment groups were stained for HDAC1 (green), COX IV (red) and DAPI (blue). Scale bar: 50 μm. High-magnification images (**Ad–Al**, scale bar: 5 μm) are demarked by white lines and are connected to the original figures **(Aa–Ac)**. Arrowheads indicate mitochondria stained for COX IV and HDAC1. **(B)** Summary showing that the number of mitochondria was dramatically decreased in TSA (25 and 50 nM)-treated animals compared to control tadpoles. *N* = 6, 5, 4 for Control, TSA 25 nM, and TSA 50 nM. **(C)** Representative co-staining images showing the anti-HDAC1 antibody (red) and MitoTracker (MTracker, green) after tadpoles were treated with TSA (50 nM, **Cb**), MS-275 (10 μM, **Cc**) and MGCD0103 (25 μM, **Cd**). Scale bar: 50 μm. High-magnification images (**Ce–Cp**, scale bar: 5 μm) are demarked by white lines from the original figures **(Ca–Cd)**. Arrowheads indicate mitochondria stained with MitoTracker and HDAC1. Scale bar: 5 μm. **(D)** Quantification showing that the number of mitochondria was dramatically decreased in TSA-, MS-275-, and MGCD0103-treated animals compared to control tadpoles. *N* = 4, 5, 5, 4 for Control, TSA, MS-275, and MGCD0103. ^*^p < 0.05, ^**^p < 0.01.

To test directly whether HDAC activity affected the COX IV expression in the mitochondria during tectal development, we exposed tadpoles to Trichostatin A (TSA, 25 or 50 nM), a broad HDAC inhibitor, in Steinberg's solution at stage 30. After 24 h, the tadpoles were fixed and co-immunostained with an anti-COX IV antibody and an anti-HDAC1 antibody (Figure 9A). We found that the number of COX IV-positive clusters was significantly reduced in the TSA (25 and 50 nM)-treated tadpoles compared to control tadpoles in a dose-dependent manner (Figure 9B).

To further test whether HDAC inhibitors regulate mitochondrial dynamics, we labeled mitochondria with the MitoTracker after exposing tadpoles in TSA (50 nM) for 24 h. The number of mitochondria was dramatically decreased in TSA-treated tadpoles compared to control tadpoles (Figures 9C,D). To test whether other HDAC family members are involved in the regulation of mitochondrial dynamics, MS-275 (10 μM) and MGCD0103 (25 μM), two specific class I HDACs inhibitors (Bolden et al., 2006; Bradner et al., 2010) were applied to the tadpole bath for 24 h. We found that the number of mitochondria was significantly decreased in either MS-275- or MGCD0103-treated tadpoles. These data indicate that the number of mitochondria are mainly regulated by class I HDACs during tectal development.

## Discussion

Our results demonstrate that in the developing tectum of *Xenopus laevis* tadpoles, the subcellular localization of class I HDACs is predominantly in the mitochondria at earlier stages and is redistributed to the nuclei at later stages. The mitochondria are accumulated in the cytoplasm with punctiform shape and are scattered with elongated phenotype over the course of brain development. At stage 37, aggregated mitochondria showed low levels of histone acetylation. Furthermore, class I HDAC inhibitors dramatically decreased the number of mitochondria. Interestingly, transient localization of class I HDACs in the mitochondria may play an important role in mitochondrial dynamics. Understanding the subcellular localization pattern of HDACs during brain development provides a valuable resource for studying the role of epigenetic regulation in mitochondrial dynamics and brain formation *in vivo*.

Histone acetylation via histone acetyltransferases (HATs) up-regulates gene expression, whereas deacetylation by histone deacetylases (HDACs) results in gene repression (Strahl and Allis, 2000). HDACs are classified into four families (class I, IIa, IIb, and IV) based on different domain structures, subcellular localization patterns, and functions (Haberland et al., 2009). Class I HDACs (HDACs 1, 2, 3, and 8) are primarily localized in the nucleus, where they regulate histone acetylation (de Ruijter et al., 2003; Broide et al., 2007). To our knowledge, however, few studies have addressed the subcellular distribution of class I HDACs in the developing brain. We used *Xenopus laevis* as a model system to study the subcellular distribution of HDACs in the developing tectum because previous studies reported the expression of the maternal HDAC (HDACm, called HDAC1 here) by Western blot during oogenesis and earlier embryogenesis (Ladomery et al., 1997). Retinal ganglion cells extend their axons to the optic tectum at stages 37–38 (Chien and Harris, 1994; Cogen and Cohen-Cory, 2000), which represents an ideal time window for studying the cellular events during the formation and refinement of neural circuits. We first observed that HDAC1 is mainly localized in the accumulated mitochondria at early stage of 34 and is redistributed to the nucleus over the course of development from stage 34 to stage 45. The transient localization of HDAC1 in the mitochondria is only maintained for a short time, from stage 34 to stage 45 (approximately 2 days). Our observations reveal that HDAC1 is not exclusively distributed in the nucleus as was previously reported (de Ruijter et al., 2003; Broide et al., 2007). The nuclear translocation of HDAC1 might be regulated by phosphorylation in sites of NLS in *Xenopus laevis* (Smillie et al., 2004) or by interaction with CRM-1 (Kim et al., 2010). However, the molecular mechanism underlying the export of HDACs from the mitochondria and the subsequent import of HDACs to the nucleus remains to be identified in detail.

We observed similar changes in the subcellular distribution for HDAC1 and HDAC2 in the developing tectum, suggesting that HDAC1 may interact with HDAC2 to form a complex (Emiliani et al., 1998; Yang and Seto, 2008). Previous studies demonstrated that HDAC3 could be predominantly located in the nucleus (Emiliani et al., 1998) or expressed both in the nucleus and the cytoplasm (Yang et al., 2002). In the developing tectum, HDAC3 is localized both in the cytoplasm and mitochondria at earlier stages and mainly expressed in the nucleus at later stages. Because the sequence of HDAC3 contains both a NES and a NLS, which determine the localization of HDAC3 (Yang et al., 2002), it suggests that the regulation of the balance of the NES and NLS may dictate the subcellular distribution of HDAC3 in the developing tectum. However, HDAC3 may also form a co-repressor complex with other HDACs that bind to DNA (Fischle et al., 2001). Unlike other class I HDACs, HDAC8 is an X-linked deacetylase and is not present in *C. elegans* or *D. melanogaster* (Yang and Seto, 2008). It is predominantly localized in the cytoplasm throughout the development of the optic tectum, suggesting that HDAC8 is specific to vertebrates. HDAC8 does not form multisubunit complexes or bind to other transcriptional cofactors (de Ruijter et al., 2003; Yang and Seto, 2008), but it can deacetylate cytoplasmic proteins, suggesting that it plays a role in cell differentiation and maturation (Tiwari et al., 2014).

The transient subcellular distribution of class I HDACs in the mitochondria may depend on the importing of nuclear-encoded proteins that are synthesized in the cytoplasm (Attardi and Schatz, 1988). Class I HDACs are nuclear-encoded proteins that are unlikely to be synthesized in the mitochondria, although transcription of mtDNA is activated in the early development of *Xenopus laevis* (Chase and Dawid, 1972). Changes in the interaction between the nucleus and mitochondria at different developmental stages may account for the transient localization of HDACs in the mitochondria (Attardi and Schatz, 1988). It is worth pursuing the mechanism underlying the transient mitochondrial expression of class I HDACs and their long-term expression in the nucleus and the effects of this expression pattern on neural circuit development (Kim et al., 2010).

The dynamics of mitochondrial morphology are regulated by the balance of fission and fusion of mitochondria. Unbalanced fission results in mitochondrial fragmentation during the early development of the tectum, whereas unbalanced fusion leads to elongation at later developmental stages (Okamoto and Shaw, 2005; Chan, 2006). Fragmented mitochondria are often found in apoptotic or neurodegenerative cells in vertebrate brains. Mitochondrial swelling and stretching is suggestive of dysfunction in apoptotic cells (Frank et al., 2001) or in Mgm1/OPA1-deficient cells (Griparic et al., 2004). However, in the critical period of tectal circuit formation, from stage 34 to stage 45, mitochondrial fragmentation is not likely due to apoptosis or to aberrant cell functions. Accumulation of spherical or ellipsoid mitochondria was often observed in cardiomyocytes (Amchenkova et al., 1988). The transformation from spherical to elongated mitochondria is paralleled by the formation of the well-defined cristae that are required for increased metabolic activity (Sathananthan and Trounson, 2000). At earlier stages of tectal development, neurites are not well developed (small neuropil, such as stage 34), and mitochondria tend to aggregate in the cell body to increase ATP production (Cheng et al., 2010), which was shown as COX IV-positive clusters in tectal cells. When the optic tract has largely innervated the optic tectum and neuropil is formed by extending complex dendrites (large neuropil, such as stage 45) (Chien and Harris, 1994), mitochondrial movement to dendrites and spines allows the efficient supply of energy in response to the metabolic demands of synapses and growth cones (Guedes-Dias and Oliveira, 2013), and this in turn plays important roles in regulating synapse density and plasticity (Li et al., 2004). When tadpoles reach stage 45, formation of a complex neural network is required to produce visually guided avoidance behavior (Shen et al., 2011, 2014).

The effect of HDAC inhibitors on apoptosis is controversial. Several studies have demonstrated that HDAC inhibitors can lead to caspase-dependent apoptosis (Cohen et al., 2004; Subramanian et al., 2005) or promote the production of reactive oxygen species (ROS) (Bolden et al., 2006) by activating the release of cytochrome c from mitochondria in cultured cells. However, some studies demonstrated that suberoylanilide hydroxamic acid (SAHA) results in mitochondrial elongation without the induction of apoptosis (Lee et al., 2012). Treatment with inhibitors of class I but not class II HDACs resulted in an increase in mitochondrial density and activity (Galmozzi et al., 2013). Our results reveal that TSA, MS-275, or MGCD0103 treatment decreases the number of mitochondria during early brain development, indicating that class I HDACs may regulate mitochondrial dynamics and metabolism (Mattson et al., 2008; Sun et al., 2014) through the apoptotic pathway. As a result, tectal cells that lack mitochondrial fusion or show increased apoptosis (Cohen et al., 2004; Subramanian et al., 2005) may lead to slower tectal development (Tao et al., 2015).

Post-transcriptional modifications of histones, including acetylation, methylation, phosphorylation, ubiquitination, and sumoylation, have been implicated in neurogenesis (Dovey et al., 2010) and plasticity (Guan et al., 2009; Gupta et al., 2010; Peixoto and Abel, 2013). HDAC family members regulate histone acetylation, such as at H4K12, to activate gene expression. Interestingly, the activity of histone acetylation is low, suggesting that the transient localization of class I HDACs may function as a transcriptional repressor to inhibit the gene expression that is correlated to the changes in mitochondrial morphology (Haberland et al., 2009). Surprisingly, the acetylation of histone in the mitochondria is very low during the tectal development, whereas the levels of acetylation in the nucleus decrease from stage 37 to stage 45. This suggests that acetylation may serve as an indicator of repression or an active marker of transcription in neurogenesis during brain development (Gupta et al., 2010). Therefore, the study of histone acetylation in the regulation of mitochondrial biogenesis is intriguing and will provide further insights into the epigenetic mechanisms that may regulate neurogenesis and neural circuit formation.

Our findings highlight the potential significance of using younger tadpoles as an ideal model system for studying mitochondria dynamics and function. The exact function of the transient localization of class I HDACs in mitochondria remains to be elucidated.

## Author contributions

All authors had full access to all the data in the study and take responsibility for the integrity of the data and the accuracy of the data analysis. Study concept and design: XG and WS. Acquisition of data: XG, HR, YT, XQ, XL, LQ, JG, and WS. Analysis and interpretation of data: XG, WS, and SD. Drafting of the manuscript: WS. Statistical analysis: XG and WS.

### Conflict of interest statement

The authors declare that the research was conducted in the absence of any commercial or financial relationships that could be construed as a potential conflict of interest.

## Supplementary material

Figure S1 **Alignment of HDAC1, HDAC2, HDAC3 and HDAC8 sequences**. Alignment of amino acid sequences from *Homo sapiens (HS)* and *Xenopus laevis (XL)*. Homology is indicated by an asterisk below aligned residues. Divergence is indicated by a blank space. Conserved sequences are indicated by a period below the aligned residues. Sequences used for generating antibodies are indicated by underlining. **(A)** Alignment of HDAC1 between HS (HDAC1_HS, accession NP_004955.2) and XL (HDAC1_1A, NP_001081491 and HDAC1_1B, NP_001079396). **(B)** Alignment of HDAC2 between HS (HDAC2_HS, NP_001518) and XL (HDAC2_XL, AAH81054). **(C)** Alignment of HDAC3 between HS (HDAC3_HS, NP_003874.2) and XL (HDAC3_XL, AAH70873). **(D)** Alignment of HDAC8 between HS (HDAC8_HS, AAF73428.1) and XL (HDAC8_XL, NP_001085711).

Figure S2 **Confirmation of antibody specificity. (A–D)** Xenopus tecta and murine brain were homogenized and immunoblotted with the anti-HDAC1 **(A)**, anti-HDAC2 **(B)**, anti-HDAC3 **(C)**, and anti-HDAC8 **(D)** antibodies. The peptide sequences used to generate the antibodies are underlined in Figure S1.

Figure S3 **Identification of cell organelles in stage 47 tectum. (A)** Representative staining images showing the colocalization of Lamp1- and COX IV-positive cluster. Zoomed in images (right) are demarked by white lines of left original images. Arrow head indicate the punctiform mitochondria that has COX IV staining but not Lamp1 staining. Arrow indicated the cluster that only has Lamp1 staining. **(B)** Representative staining images showing the colocalization of EEA1- and COX IV-positive cluster. Arrow head indicate the cluster that has COX IV staining but not Lamp1 staining. Arrow indicated the cluster that only has EEA1 staining. **(C)** Representative staining images showing the colocalization of Calnexin- and COX IV-positive cluster. Arrow head indicate the cluster that has COX IV staining but not Lamp1 staining. Arrow indicated the cluster that only has Calnexin staining. Scale bar: 50 μm. Zoomed in scale bar: 5 μm.
