# Supplementary material for: Subcellular Localization of Class I Histone Deacetylases in the Developing Xenopus tectum
Source: Front Cell Neurosci. 2016 Jan 12;9:510. doi: 10.3389/fncel.2015.00510 (PMC4709447; doi:10.3389/fncel.2015.00510)
Supplement: Figure S1 — Alignment of HDAC1, HDAC2, HDAC3 and HDAC8 sequences. Alignment of amino acid sequences from Homo sapiens (HS) and Xenopus laevis (XL). Homology is indicated by an asterisk below aligned residues. Divergence is indicated by a blank space. Conserved sequences are indicated by a period below the aligned residues. Sequences used for generating antibodies are indicated by underlining. (A) Alignment of HDAC1 between HS (HDAC1_HS, accession NP_004955.2) and XL (HDAC1_1A, NP_001081491 and HDAC1_1B, NP_001079396). (B) Alignment of HDAC2 between HS (HDAC2_HS, NP_001518) and XL (HDAC2_XL, AAH81054). (C) Alignment of HDAC3 between HS (HDAC3_HS, NP_003874.2) and XL (HDAC3_XL, AAH70873). (D) Alignment of HDAC8 between HS (HDAC8_HS, AAF73428.1) and XL (HDAC8_XL, NP_001085711). [file FigureS1.docx]

**Sup Fig 1**

1. **HDAC1 alignment between *Homo Sapiens* (HDAC1_HS) and *Xenopus laevis*(HDAC1_1A and HDAC1_1B) (**Identity: 90.46%)

HDAC1_HS MAQTQGTRRKVCYYYDGDVGNYYYGQGHPMKPHRIRMTHNLLLNYGLYRKMEIYRPHKAN

HDAC1_1A MALTLGTKKKVCYYYDGDVGNYYYGQGHPMKPHRIRMTHNLLLNYGLYRKMEIFRPHKAS

HDAC1_1B MALSQGTKKKVCYYYDGDVGNYYYGQGHPMKPHRIRMTHNLLLNYGLYRKMEIYRPHKAS

** . **..********************************************.*****

HDAC1_HS AEEMTKYHSDDYIKFLRSIRPDNMSEYSKQMQRFNVGEDCPVFDGLFEFCQLSTGGSVAS

HDAC1_1A AEDMTKYHSDDYIKFLRSIRPDNMSEYSKQMQRFNVGEDCPVFDGLFEFCQLSAGGSVAS

HDAC1_1B AEEMTKYHSDDYIKFLRSIRPDNMSEYSKQMQRFNVGEDCPVFDGLFEFCQLSTGGSVAS

**.**************************************************.******

HDAC1_HS AVKLNKQQTDIAVNWAGGLHHAKKSEASGFCYVNDIVLAILELLKYHQRVLYIDIDIHHG

HDAC1_1A AVKLNKQQTDISVNWSGGLHHAKKSEASGFCYVNDIVLAILELLKYHQRVVYIDIDIHHG

HDAC1_1B AVKLNKQQTDISVNWSGGLHHAKKSEASGFCYVNDIVLAILELLKYHQRVVYIDIDIHHG

***********.***.**********************************.*********

HDAC1_HS DGVEEAFYTTDRVMTVSFHKYGEYFPGTGDLRDIGAGKGKYYAVNYPLRDGIDDESYEAI

HDAC1_1A DGVEEAFYTTDRVMTVSFHKYGEYFPGTGDLRDIGAGKGKYYAVNYALRDGIDDESYEAI

HDAC1_1B DGVEEAFYTTDRVMSVSFHKYGEYFPGTGDLRDIGAGKGKYYAVNYPLRDGIDDESYEAI

**************.******************************* *************

HDAC1_HS FKPVMSKVMEMFQPSAVVLQCGSDSLSGDRLGCFNLTIKGHAKCVEFVKSFNLPMLMLGG

HDAC1_1A FKPVMSKVMEMFQPSAVVLQCGADSLSGDRLGCFNLTIKGHAKCVEFIKTFNLPLLMLGG

HDAC1_1B FKPVMTKVMEMFQPSAVVLQCGADSLSGDRLGCFNLTIKGHAKCVEFIKTFNLPMLMLGG

*****.****************.************************.*.****.*****

HDAC1_HS GGYTIRNVARCWTYETAVALDTEIPNELPYNDYFEYFGPDFKLHISPSNMTNQNTNEYLE

HDAC1_1A GGYTIRNVARCWTYETAVALDSEIPNELPYNDYFEYFGPDFKLHISPSNMTNQNTNEYLE

HDAC1_1B GGYTIRNVARCWTYETAVALDSEIPNELPYNDYFEYFGPDFKLHISPSNMTNQNTNEYLE

*********************.**************************************

HDAC1_HS KIKQRLFENLRMLPHAPGVQMQAIPEDAIPEESGDEDEDDPDKRISICSSDKRIACEEEF

HDAC1_1A KIKQRLFENLRMLPHAPGVQMQAVAEDSIHDDSGEEDEDDPDKRISIRSSDKRIACDEEF

HDAC1_1B KIKQRLFENLRMLPHAPGVQMQAIPEDSVHDDSGEEDEEDPDKRISIRSSDKRIACDEEF

***********************. **.. ..**.***.******** ********.***

HDAC1_HS SDSEEEGEGGRKNSSNFKKAKRVKTEDEKEKDPEEKKEVTEEEKTKEEKPEAKGVKEEVKLA

HDAC1_1A SDSEDEGEGGRKNVANFKKVKRVKTEEEKEG--EDKKDVKEEEKAKDEKTDSKRVKEETKSV

HDAC1_1B SDSEDEGEGGRKNVANFKKVKRVKTEEEKEG--EDKKDVKEEEKAKDEKTDSKRVKEETKSV

****.******** .**** ******.*** *.**.* ****.*.** ..* **** *

1. **HDAC2 alignment between *Homo Sapiens* (HDAC2_HS) and *Xenopus laevis* (HDAC2_XL) (**Identity: 97.13%)

HDAC2-HS MAYSQGGGKKKVCYYYDGDIGNYYYGQGHPMKPHRIRMTHNLLLNYGLYRKMEIYRPHKA

HDAC2-XL MAYTQGGAKKKVCYYYDGDIGNYYYGQGHPMKPHRIRMTHNLLLNYGLYRKMEIYRPHKA

***.*** ****************************************************

HDAC2-HS TAEEMTKYHSDEYIKFLRSIRPDNMSEYSKQMQRFNVGEDCPVFDGLFEFCQLSTGGSVA

HDAC2-XL TAEEMTKYHSDEYIKFLRSIRPDNMSEYSKQMQRFNVGEDCPVFDGLFEFCQLSTGGSVA

************************************************************

HDAC2-HS GAVKLNRQQTDMAVNWAGGLHHAKKSEASGFCYVNDIVLAILELLKYHQRVLYIDIDIHH

HDAC2-XL GAVKLNRQQTDMAVNWAGGLHHAKKSEASGFCYVNDIVLGILELLKYHQRVLYIDIDIHH

*************************************** ********************

HDAC2-HS GDGVEEAFYTTDRVMTVSFHKYGEYFPGTGDLRDIGAGKGKYYAVNFPMRDGIDDESYGQ

HDAC2-XL GDGVEEAFYTTDRVMTVSFHKYGEYFPGTGDLRDIGAGKGKYYAVNFPMRDGIDDESYGQ

************************************************************

HDAC2-HS IFKPIISKVMEMYQPSAVVLQCGADSLSGDRLGCFNLTVKGHAKCVEVVKTFNLPLLMLG

HDAC2-XL IFKPIISKVMEMYQPSAVVLQCGADSLSGDRLGCFNLTVKGHAKCVEVVKTFNLPLLMLG

************************************************************

HDAC2-HS GGGYTIRNVARCWTYETAVALDCEIPNELPYNDYFEYFGPDFKLHISPSNMTNQNTPEYM

HDAC2-XL GGGYTIRNVARCWTYETAVALDCEIPNELPYNDYFEYFGPDFKLHISPSNMTNQNTPEYM

************************************************************

HDAC2-HS EKIKQRLFENLRMLPHAPGVQMQAIPEDAVHEDSGDEDGEDPDKRISIRASDKRIACDEE

HDAC2-XL EKIKQRLFENLRMLPHAPGVQMQAIPEDAVQEDSGDEEGEDPDKRISIRASDKRIACDEE

******************************.******.**********************

HDAC2-HS FSDSEDEGEGGRRNVADHKKGAKKARIEEDKKETEDKKTDVKEEDKSKDNSGEKTDTKGT

HDAC2-XL FSDSEDEGEGGRRNVADHKKGAKKARLEEDKKETDDKKSDVKEEDKSKDNIAEKMDTKGV

**************************.*******.***.*********** ** ****

HDAC2-HS KSEQLSNP

HDAC2-XL KSEQPINP

**** **

1. **HDAC3 alignment between *Homo Sapiens* (HDAC3_HS) and *Xenopus laevis*(HDAC3_XL) (**Identity: 94.86%)

HDAC3-HS MAKTVAYFYDPDVGNFHYGAGHPMKPHRLALTHSLVLHYGLYKKMIVFKPYQASQHDMCR

HDAC3-XL MAKTVAYFYDPDVGNFHYGTGHPMKPHRLSLTHSLVLHYGLYKKMIVFKPYQASQHDMCR

*******************.*********.******************************

HDAC3-HS FHSEDYIDFLQRVSPTNMQGFTKSLNAFNVGDDCPVFPGLFEFCSRYTGASLQGATQLNN

HDAC3-XL FHSEDYIDFLQRVSPNNMQGFTKSLNAFNVGDDCPVFPGLFEFCSRYTGASLQGATLLNN

***************.**************************************** ***

HDAC3-HS KICDIAINWAGGLHHAKKFEASGFCYVNDIVIGILELLKYHPRVLYIDIDIHHGDGVQEA

HDAC3-XL KNCDIAINWAGGLHHAKKFEASGFCYVNDIVIGILELLKYHPRVLYIDIDIHHGDGVQEA

* **********************************************************

HDAC3-HS FYLTDRVMTVSFHKYGNYFFPGTGDMYEVGAESGRYYCLNVPLRDGIDDQSYKHLFQPVI

HDAC3-XL FYLTDRVMTVSFHKYGNYFFPGTGDMYEVGAESGRYYCLNVPLRDGIDDQSYRHLFQPVI

****************************************************.*******

HDAC3-HS NQVVDFYQPTCIVLQCGADSLGCDRLGCFNLSIRGHGECVEYVKSFNIPLLVLGGGGYTV

HDAC3-XL KQVIDFYQPTCIVLQCGADSLGCDRLGCFNLSIRGHGECVQYVKSFNIPLLVLGGGGYTV

**.************************************.*******************

HDAC3-HS RNVARCWTYETSLLVEEAISEELPYSEYFEYFAPDFTLHPDVSTRIENQNSRQYLDQIRQ

HDAC3-XL RNVARCWTYETSLLVDETISEELPYSEYFEYFAPDFTLHPDVSTRIENQNSRQYLDQIRQ

***************.*.******************************************

HDAC3-HS TIFENLKMLNHAPSVQIHDVPADLLTYDRTDEADAEERGPEENYSRPEAPNEFYDGDHDN

HDAC3-XL TVFESLKMLNHAPSVQIHDVPSDILNYERTDEPDPEERGGEDNYSRPEASNEFYDGDHDN

*.** ****************.*.*.*.**** * **** *.******* **********

HDAC3-HS DKESDVEI

HDAC3-XL DKESDVEI

********

1. **HDAC8 alignment between *Homo Sapiens* (HDAC8_HS) and *Xenopus laevis*(HDAC8_XL) (**Identity: 70.82%)

HDAC8-HS MEEPEEPADSGQSLVPVYIYSPEYVSMCDSLAKIPKRASMVHSLIEAYALHKQMRIVKPK

HDAC8-XL ----------------------------------------------------MSRVVKPK

*.****

HDAC8-HS VASMEEMATFHTDAYLQHLQKVSQEGDDDHPDSIEYGLGYDCPATEGIFDYAAAIGGATI

HDAC8-XL VASMEEMAAFHTDAYLQHLHKVSEEGDNDDPETLEYGLGYDCPITEGIYDYAAAVGGATL

********.**********.***.*** * *...********* ****.*****.****.

HDAC8-HS TAAQCLIDGMCKVAINWSGGWHHAKKDEASGFCYLNDAVLGILRLRRKFERILYVDLDLH

HDAC8-XL TAAEQLIEGKTRIAVNWPGGWHHAKKDEASGFCYLNDAVLGILKLREKFDRVLYVDMDLH

***. **.* ...*.** *************************.** **.*.****.***

HDAC8-HS HGDGVEDAFSFTSKVMTVSLHKFSPGFFPGTGDVSDVGLGKGWYYSVNVPIQDGIQDEKY

HDAC8-XL HGDGVEDAFSFTSKVMTVSLHKFSPGFFPGTGDVSDIGLGKGRYYSINVPLQDGIQDDKY

************************************.***** ***.***.******.**

HDAC8-HS YQICESVLKEVYQAFNPKAVVLQLGADTIAGDPMCSFNMTPVGIGKCLKYILQWQLATLI

HDAC8-XL YQICEGVLKEVFTTFNPEAVVLQLGADTIAGDPMCSFNMTPEGIGKCLKYVLQWQLPTLI

***** *****. .*** *********************** ********.***** ***

HDAC8-HS LGGGGYNLANTARCWTYLTGVILGKTLSSEIPDHEFFTAYGPDYVLEITPSCRPDRNEPH

HDAC8-XL LGGGGYHLPNTARCWTYLTALIVGRTLSSEIPDHEFFTEYGPDYVLEITPSCRPDRNDTQ

******.* ********** .*.*.************* ******************. .

HDAC8-HS RIQQILNYIKGNLKHVV

HDAC8-XL KVQEILQSIKGNLKRVV

..*.**. ******.**
