# Supplementary figures and images for: Subcellular Localization of Class I Histone Deacetylases in the Developing Xenopus tectum
Source: Front Cell Neurosci. 2016 Jan 12;9:510. doi: 10.3389/fncel.2015.00510 (PMC4709447; doi:10.3389/fncel.2015.00510)

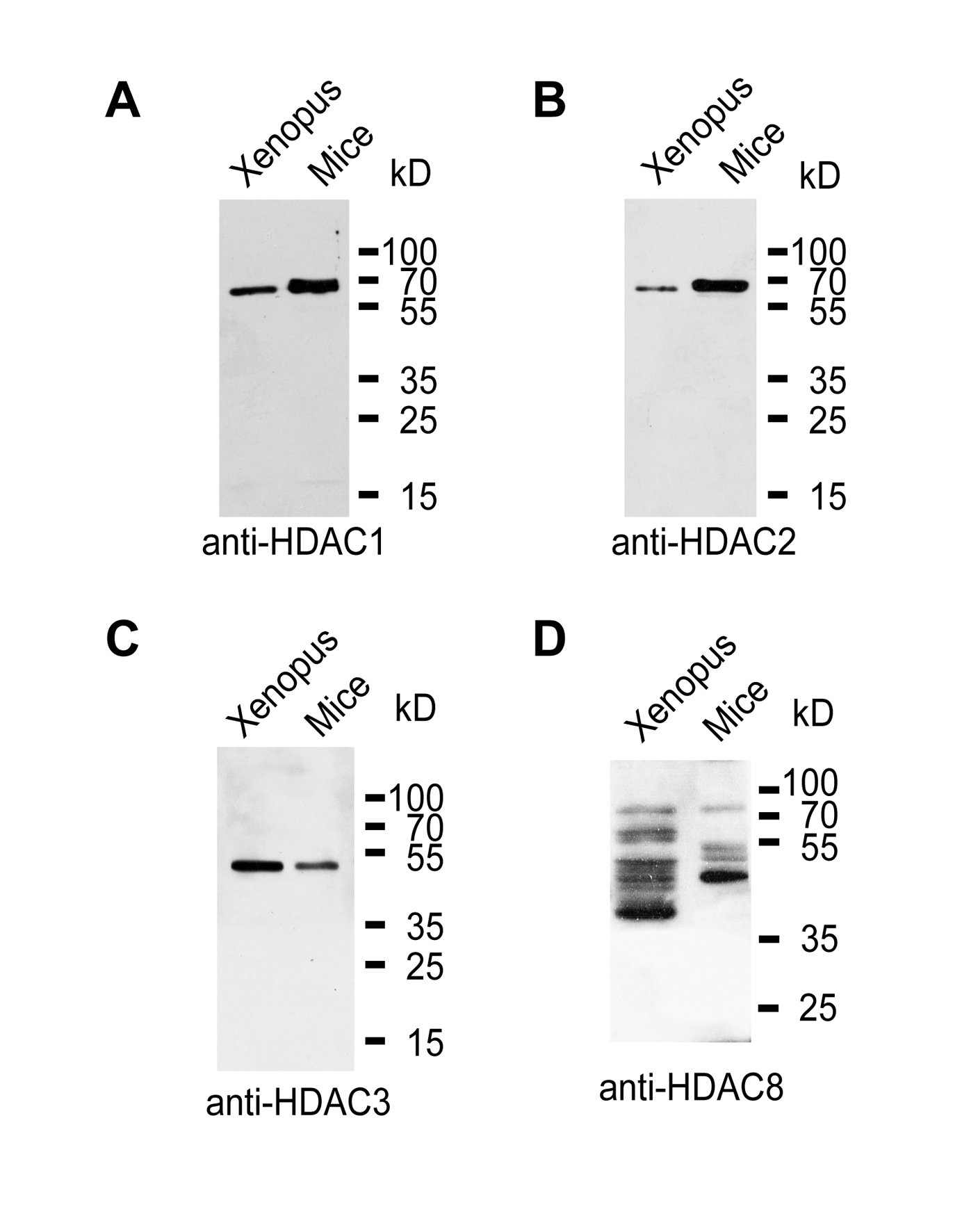

Supplement: Figure S2 — Confirmation of antibody specificity. (A–D) Xenopus tecta and murine brain were homogenized and immunoblotted with the anti-HDAC1 (A), anti-HDAC2 (B), anti-HDAC3 (C), and anti-HDAC8 (D) antibodies. The peptide sequences used to generate the antibodies are underlined in Figure S1. [file FigureS2.tif]

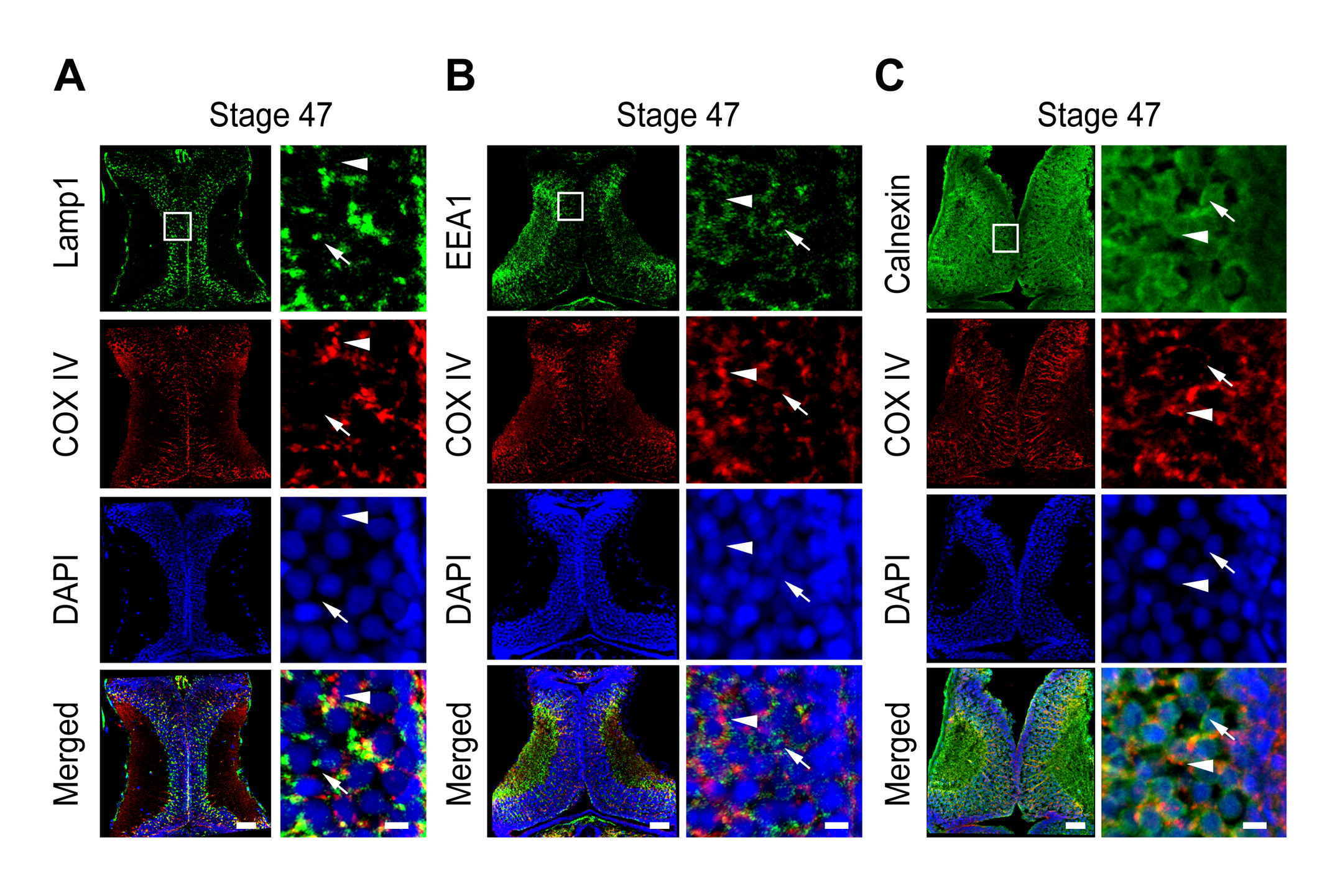

Supplement: Figure S3 — Identification of cell organelles in stage 47 tectum. (A) Representative staining images showing the colocalization of Lamp1- and COX IV-positive cluster. Zoomed in images (right) are demarked by white lines of left original images. Arrow head indicate the punctiform mitochondria that has COX IV staining but not Lamp1 staining. Arrow indicated the cluster that only has Lamp1 staining. (B) Representative staining images showing the colocalization of EEA1- and COX IV-positive cluster. Arrow head indicate the cluster that has COX IV staining but not Lamp1 staining. Arrow indicated the cluster that only has EEA1 staining. (C) Representative staining images showing the colocalization of Calnexin- and COX IV-positive cluster. Arrow head indicate the cluster that has COX IV staining but not Lamp1 staining. Arrow indicated the cluster that only has Calnexin staining. Scale bar: 50 μm. Zoomed in scale bar: 5 μm. [file FigureS3.tif]
